# Contextual Association between Political Regime and Adolescent Suicide Risk in Korea: A 12-year Repeated Cross-Sectional Study from Korea

**DOI:** 10.3390/ijerph16050874

**Published:** 2019-03-10

**Authors:** Sang Jun Eun

**Affiliations:** Department of Preventive Medicine, Chungnam National University College of Medicine, Daejeon 35015, Korea; zepplin7@cnu.ac.kr; Tel.: +82-42-580-8262

**Keywords:** politics, attempted suicide, depression, adolescent health, multilevel analysis

## Abstract

This study evaluated associations between contextual political determinants and individual adolescent suicide risk (SR). Using repeated cross-sectional individual-level data of 829,861 students in the Korea Youth Risk Behavior Web-based Survey and national contextual-level data during 2005–2016, cross-classified random effects models were conducted to estimate fixed period and cohort effects of political determinants on SR. Adolescent SR was reduced during conservative presidential regimes. Contrary to presidencies’ period effects, conservative regimes had negative cohort effects on adolescent SR. The odds of suicide attempt and depression increased in the grade cohorts affected by college entrance examination policies of conservative regimes. Politics has significantly impacted adolescent SR despite differences in period and cohort effects of politics. These findings imply the need to encourage adolescents’ political participation in choosing political forces with policies favorable to their own mental health.

## 1. Introduction

Suicide is a leading cause of death globally and a major contributor to disability-adjusted life-years in adolescent populations, especially among high-income Asian Pacific countries [1]. The suicide rate in the Republic of Korea has been the highest among Organization for Economic Co-operation and Development countries since 2003 [2] and is the fourth highest in the world [3]. In Korea, suicide is the most common cause of youth death, and the declining trend in suicide rates among adolescents aged 10–19 years starting in 2011 has recently rebounded [4]. 

Many studies have reported various individual and contextual factors affecting suicide risk (SR). Individual risk factors include sex, age, ethnicity, marital status, educational attainment, employment, religious beliefs, health behavior (e.g., smoking, alcohol intake), mental comorbidity (e.g., depression, drug-related disorders), physical comorbidity (e.g., cancer, HIV/AIDS), heredity, sexual orientation, childhood abuse, and prior suicide attempts [5,6,7]. Contextual determinants that cover social (e.g., social isolation, exposure to suicide), economy (e.g., economic downturn, employment rate), climatological (e.g., solar exposure, precipitation), policy (e.g., availability to lethal means), and political (e.g., political regimes) factors have also been documented [5,6,7,8].

Although suicide is a complex phenomenon influenced by multilevel and multifaceted factors [9], few studies have examined the contextual factors in adolescent SR, such as economy, residential area, and social support, with adjustment for individual factors [10,11,12]. Furthermore, politics may impact health by improving living conditions through economic growth, protecting population health against health risks through social security, education or housing policies, or directly providing public health and healthcare services [13]. Some studies have shown that conservative political power was associated with increased suicide rates [8,14,15]. However, those studies were conducted in Western general populations and mostly used ecological study designs, so the contextual effects of political determinants on adolescent SR have not been demonstrated while considering individual factors.

This study aimed to investigate associations between contextual political determinants and individual adolescent SR in Korea during 2005–2016. SR comprises suicide attempt and depressive symptom, which are the most influential predictors for suicide [5,6,7]. Political determinants were changes in presidency as a period effect factor and college entrance examinations (CEE) as cohort effect factors. Korea has a highly-centralized presidential system, where the political power of parliament and, regional or local governments is weak and overwhelmed by that of the president [16,17]. The college admission policy strongly affects Korea’s entire education system, because success in life and careers can almost be determined by CEE scores [18]. Educational policy such as the CEE system may have a potent impact on adolescents’ lives, most of whom are students, and has been crucially influenced by political regime changes [18]. Analysis was adjusted for potential confounders at individual and contextual levels.

## 2. Materials and Methods 

### 2.1. Data Sources

This study used the Korea Youth Risk Behavior Web-based Survey (KYRBS) data from the Korea Centers for Disease Control and Prevention, which has been conducted annually since 2005 [19]. KYRBS were nationally representative, cross-sectional, self-reported samples of middle- and high-school students collected on various health-related behaviors [19]. The twelfth-grade students in 2005 were not surveyed owing to the College Scholastic Ability Test (CSAT), the Korean national CEE [19]. KYRBS data were two-stage stratified cluster random samples (school and classroom), but only the sample weights at student level were publicly released. During 2005–2016, school participation rates ranged from 99.6% to 100.0% and student response rates were 89.7% to 97.7% (average 95.4%). Of 855,763 participants in the 2005–2016 KYRBS, excluding 25,902 who did not respond to study variables, 829,861 (97.0%) students were included in the analysis. For the level-2 contextual variables, publicly available aggregated data were used from the Statistics Korea (national statistical office in Korea) and Supreme Prosecutors’ Office website [20,21].

### 2.2. Outcomes

Suicide risks consisted of suicide attempt and depressive symptom, measured as positive responses to the following questions: “In the last 12 months, have you ever attempted suicide?” and “In the last 12 months, have you experienced sadness or hopelessness that interfered with your everyday life for at least two weeks?”

### 2.3. Political Factors

**Figure 1:**
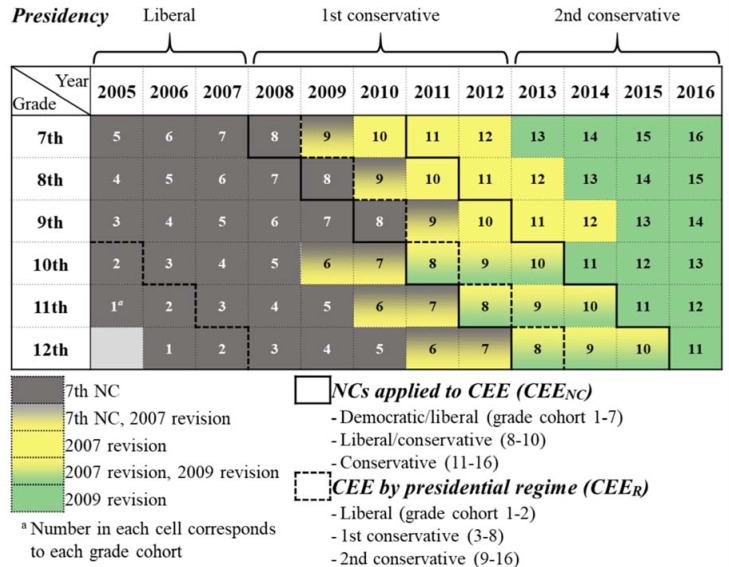
Political determinants and major suicide prevention policies in Korea. CEE, college entrance examination; NC, National Curriculum.

Political determinants were changes in presidency and CEE. As a period-effect variable, presidency was measured as liberal (President Moo-hyun Roh, 2005–2007, whose presidential term began in 2003), first conservative (Myung-bak Lee, 2008–2012), and second conservative (Geun-hye Park, 2013–2016) regimes divided by presidential term (Figure 1). Suicide prevention policies for each regime [22,23] are presented in Supplementary Figure S1.

The Korean people have traditionally put tremendous value on education, especially entrance into prestigious universities, because a good academic background has been crucial to social mobility and success in the labor market, despite not guaranteeing either [18,24]. Therefore, as most students in secondary, or even preschool to primary education, work fiercely to achieve high scores in exams, often called “exam hell”, CSAT has been the most powerful factor in the Korean education system [18]. Accordingly, each regime implemented policies to mitigate the CEE system’s overreliance on CSAT. Supplementary Table S1 describes CEE policies implemented by each regime [18,25,26]. Major changes have been made to the CEE system mainly due to amendment of the National Curriculum (NC) and presidential regime change [18,24,27]. CEE_NC_, the first cohort effect variable, was categorized into three groups depending on the NCs reflected in CEE by grade cohorts: democratic/liberal (grade cohort 1–7: CEE applying the 7th NC and 2007 revision), liberal/conservative (grade cohort 8–10: 2007 revision, 2009 revision), and conservative (grade cohort 11–16: 2009 revision) (Figure 1 and Supplementary Table S1). CEE_R_, the second cohort effect variable, was defined as liberal (grade cohort 1–2), first conservative (grade cohort 3–8), and second conservative (grade cohort 9–16) according to the presidential regime establishing the CEE system for which each grade cohort applied (Figure 1).

### 2.4. Covariates

Among all available variables at individual [19] and contextual level [20,21], explanatory variables investigated for all study years were selected. Contextual covariates consisted of economic (real household final consumption expenditure per capita and percentage change of house price index), social (college enrolment rate and adolescent crime rate), and income inequality factors (labor income share) [20,21]. Because political factors were collinear and correlated with other covariates, especially economic factors, various combinations among all available covariates possibly relevant to SR were tested for multicollinearity. Then, covariates were selected that minimized the risk of multicollinearity (variance inflation factor [VIF] <10, condition index <30). Maximum VIF was 7.7 for presidency, with a condition index of 13.9. Individual covariates consisted of sociodemographic (age, sex, academic performance, household economic status, and residential area), health behavioral (tobacco use, alcohol use, vigorous physical activity, body mass index, healthy and unhealthy diet), and mental health factors (stress level, sleep sufficiency, and suicidal thought), all of were based on self-reported responses [19]. As questionnaire response codes have changed every several years, they were recoded to indicate the same condition consistently. VIFs for individual covariates were all less than 2 (maximum VIF was 1.4 for alcohol use). Supplementary Table S2 describes covariates.

### 2.5. Analyses

Descriptive statistics of the 2005–2016 KYRBS data were analyzed and national representative prevalence of SR was estimated using survey weights [28]. Repeated cross-sectional study design was adopted using a hierarchical age–period–cohort cross-classified random-effects model, which estimates the contextual effects of interest while controlling covariates at all levels [29]. An intercept-only model was estimated to test whether students’ SR varied by both 16-grade cohorts according to the first of year 7th grade and over a 12-year period (Figure 1), and intra-class correlations (ICCs) were calculated. Cross-classified random intercept logistic regression models were run using the Laplace approximation and Cholesky method to evaluate political determinants’ fixed effects on suicide attempt and depressive symptom while controlling for random effects of grade cohorts and periods and fixed effects of individual (age, sex, academic performance, household economic status, residential area, tobacco use, alcohol use, vigorous physical activity, body mass index, healthy and unhealthy diet, stress level, sleep sufficiency, and suicidal thought) and contextual covariates (real household final consumption expenditure per capita, percentage change of house price index, college enrolment rate, adolescent crime rate, and labor income share) (Supplementary Table S2). Time lags for the effects of political and contextual predictors were not assumed, because models fitted better when no time lag was considered. Continuous covariates were grand-mean centered and categorical covariates were treated as dummy variables. Unweighted data were analyzed because weighting methods for cross-classified multilevel models with complex survey data have not been established [28]. The best models were selected based on the smallest Akaike’s information criterion values. The analyses were performed using ‘proc glimmix’ in SAS 9.4 (SAS Institute, Inc., Cary, NC, USA). This study was exempted from approval by the Institutional Review Board of Chungnam National University (IRB No. 201803-SB-038-01).

## 3. Results

### 3.1. Descriptive Statistics

**Figure 2:**
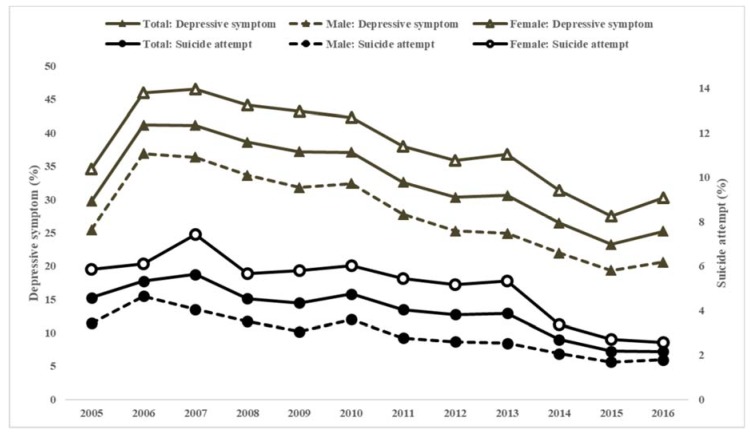
Trends in proportions of suicide attempts and depressive symptoms among Korean middle- and high-school students.

For 12 years, 31% of middle- and high-school students experience depressive symptom and 4% attempted suicide. SR has gradually decreased since 2008 despite rebounding in 2016 (Figure 2). The differences in SR among individual covariates are depicted in Table 1 (all p’s <0.001). Students who were female, poor academic performers, less wealthy, residing in the capital area, or had poor health behavior and mental health status were more likely to have SR. Proportions of SR were lowest in students with obesity than any other range of body mass index. While depressive symptom increased with age, proportions of suicide attempt decreased. Students who engaged in vigorous physical activities more frequently reported less depressive symptom, but attempted suicide more often. The results for male and female students are shown in Supplementary Tables S3 and S4, which are similar to those for the total students.

### 3.2. Intercept-Only Models

In intercept-only models, suicide attempt and depressive symptom at student level varied significantly across grade cohorts and periods. As for suicide attempt, intra-period correlation was 14.3% (95% confidence interval [CI] 9.4%–18.3%) and intra-cohort correlation was 9.5% (95% CI 8.0%–17.0%). ICCs for depressive symptom were 4.9% (95% CI 3.1%–6.4%) and 10.7% (95% CI 8.3%–17.1%), respectively.

### 3.3. Cross-Classified Random Effects Models

As shown in Table 2, SR was significantly lower in the period when conservative regimes were in power than in the liberal government period. Compared to the liberal government, suicide attempt declined by 72% and 32% during the first and second conservative regimes, respectively (odds ratio [OR] 0.58 and 0.76). Depressive symptom also decreased by 42% and 43% during each conservative government period (OR 0.71 and 0.70). The cohort effect of politics was contrary to the period effect. There were more suicide attempts and depressive symptoms among middle- and high-school students who had undergone CEE on NCs amended by the conservative regimes rather than democratic/liberal governments (OR 2.51 and 1.15, respectively). Compared to students having taken the CEE instituted by the liberal government, those who took CEE set up by conservative governments were more likely to attempt suicide (OR 1.77 for the second conservative regime) and have depressive symptom (OR 1.1 for the first conservative regime and 1.15 for second one).

A one million KRW (about 910 USD) increase in real household final consumption expenditure per capita resulted in a 60% reduction in risk for suicide attempts (OR 0.62). A one percent increase in house price index lead to a 1% increase in risk for suicide attempt and 2% increase for depressive symptom. The likelihoods of suicide attempt (OR 1.07) and depressive symptom (OR 1.14) increased with college enrollment rate. A one percent increase in adolescent crime rates or labor income shares resulted in a 10% increase (OR 1.10) or 12% decrease (OR 0.89) in suicide attempt, respectively. Associations between individual-level covariates and SR were similar to patterns in descriptive statistics, although likelihood of SR increased with weekly frequency of vigorous physical activity.

## 4. Discussion

This study examined associations between contextual political determinants and individual SR in adolescents in Korea using 12 years of repeated cross-sectional individual-level data and national contextual-level data. Changes in both presidency and CEE were associated with SR, which decreased during the conservative governments’ period, while it increased under CEE influenced by conservative regimes.

These results seem to be inconsistent with previous findings that conservative regimes were associated with poorer population health, including suicide rate, which might have been attributed to lack of the government’s responsibility for health [8,13,14,30]. In Korea, however, major suicide prevention policies were on track during the first conservative administration, although the previous liberal government laid the foundations for suicide prevention policies (Supplementary Figure S1). The relatively active implementation of suicide prevention policies in the conservative regime could be regarded as a response to the surge in the suicide rate, which had rebounded since the early 2000s, during the 2008–2009 financial crisis, given intensifying social insecurity stemming from the conservative regime’s emphasis on competitiveness and market-centeredness [31]. The general election and presidential election, scheduled for April and December 2012, also had become one of the driving forces for accelerating policy implementation to reduce suicide rates [23]. School-based mental health programs were set up following the critical moments of the Virginia Tech shooting in 2007 and suicide outbreak among victims of bullying beginning in December 2011 [23]. Even though the effectiveness of those suicide prevention policies on SR was not evaluated due to the inability to disentangle beneficiaries of policies from data, and the possibility of collinearity with presidency variable, reduction of adolescent SR under the conservative regimes is partially presumed to be the outcome of suicide prevention policies [32].

Unlike presidency, CEE systems affected by conservative administrations were associated with exacerbation of adolescent SR. Notwithstanding contributions to educational equity through expansion of affirmative action for the disadvantaged, Roh’s liberal administration may not be free from responsibility for problems with the current CEE system by settling high-school records, college-administered exams, and CSAT as three elements of the CEE system, the so-called “triangle of death” [26]. In response to Roh’s policy of reducing the importance of CSAT and increasing the weight of high-school grades to alleviate excessive dependence on private education to improve CSAT scores, colleges increased the proportion of college-administered exams in CEE to select excellent students [25,26]. This resulted in an increase in students’ academic burden instead of the expected policy effects because, along with preparation for CSAT, they additionally had to improve high-school grades throughout all high-school years and prepare for college-administered exams [25,26]. The first conservative government fueled students’ academic burden by granting colleges greater autonomy in CEE [26]. Although an admission officer system was piloted in 2007 to provide opportunities for college admission to students with various potentials other than high test scores, colleges created diverse types of CEE that were advantageous for selecting outstanding students, as the conservative Lee government expanded the admission officer system [25,26]. This led to the explosion of CEE types, with more than 3000 types [26]. However, it was beyond individual schools’ ability to equip students with various competencies required for college entrance, so students’ burden was redoubled by preparation for numerous types of CEE by inclining more toward private education [25,26]. The skyrocketing complexity of CEE worked favorably for the upper class, who could afford to prepare for diverse CEEs, which in turn aggravated educational inequality [26]. Also, increased college admission corruption due to the subjectivity and opacity of the admission officer system made the CEE system be perceived as unfair [26]. The subsequent conservative government attempted to simplify CEE types, but failed to reduce the complexity of CEE, because it was merely a categorization of various CEE types [26]. Moreover, since the Park administration just changed the name of the admission officer system to the comprehensive high school record and further increased its weight in CEE, the burden of students has added up and educational inequality and unfairness has deepened [26].

NC revised in the first conservative regime allowed schools autonomy to organize their individual curricula. Instead of a fixed grade-specific curriculum according to NC, secondary schools have been able to determine their own curricula by constructing three-year courses with the subjects required for grades clusters and by either increasing or decreasing class hours of certain subjects by 20% [24]. Contrary to the policy intent of relieving learning burden and securing time for competency learning, schools increased class hours in three critically important subjects in CEE (Korean language, English, and mathematics) at the expense of diminishing hours in unimportant subjects [24]. School education focusing on preparation for CEE intensified competition among students for college admission [18,25], which would have put more stress on students. As above, increased SR in the grade cohorts affected by the CEE policies of conservative regimes might be regarded as the result of increased academic burden and intensified competition.

Associations between SR and covariates at individual and contextual levels were generally similar to previous studies [5,6,7,8,33,34,35]. These results show that improving financial affordability of households, maintaining appropriate housing prices, increasing labor income share, and preventing adolescent crime might reduce adolescent SR. Increases in SR with increased college enrollment rates might be the result of excessive competition for college entrance. Almost 70% of Korea’s college-age population has been enrolled in college [20], but college-wage premiums have not declined, which means that college graduates still earn higher wages than secondary-school graduates, and prestigious university graduates have even higher wages than non-prestigious college graduates [36]. As more students enter college, future income is likely to decrease for those who fail to be admitted to college. Intensification of competition for college admission is inevitable for differentiation and necessary for not being left behind in the labor market. This might indicate that employment policies based on job competencies regardless of academic background could help reduce adolescent SR.

SR increased in students whose household income levels were both higher and lower, which could be due to the academic burden on students from high-income families who can afford private tutoring and the fear of falling behind in the labor market for low-income families’ students [26]. Students in rural areas were more likely to attempt suicide than those in urban areas, but less likely to have depressive symptom. These results are supported by findings that adolescent suicide rates in Korean rural areas have been higher than in urban areas [20] and that psychiatric disorders were less prevalent among suicide victims in rural Chinese adolescents [7,9]. Despite the well-known protective effects of physical activity on mental health, SR increased with frequency of vigorous physical activity [37]. This might be because intimate relationships among students who frequently engage in vigorous physical activity may increase the chances of unhealthy behavior such as smoking or drinking after physical activity, resulting in increase in SR [37,38]. Underweight students had a lower SR than normal-weight students, which might be due to association between inappropriate weight control behavior and underweight [39].

This study has several limitations. First, other than suicide prevention policies or CEE, there might be another explanation for period and cohort effects of political determinants. However, no plausible event was found to explain those effects during 2005–2016. Second, moderation and mediation effects of contextual covariates in the relationship between political predictors and SR could not be investigated and heterogeneity between schools or provinces could not be taken into consideration, because models were not fitted due to the small number of clusters. Third, although suicidal thoughts and elevated stress levels could be considered SR in addition to suicide attempt and depressive symptom, models to evaluate associations between political determinants and them also did not fit, because the random effects of grade cohorts and periods were too small to be analyzed. Also, gender differences in the association between independent factors including political factors and suicide risk could not be analyzed for the same reason. Fourth, even with efforts to minimize multicollinearity risk, VIF of presidency was as high as 7.7, so multicollinearity might not have been completely avoided. Fifth, 12th grade students in 2005 were not surveyed. This might have no impact on grade cohort effect, but could have influenced period effect in the direction of overestimating prevalence of suicide attempt and underestimating that of depressive symptom in 2005, since they were the least likely to attempt suicide and most likely to have depressive symptom. Sixth, the use of self-reported data might have introduced the possibility of bias. However, self-reports have been claimed to yield more valid information than other methods on sensitive issues such as suicidal behavior [40]. Seventh, the validity of KYRBS data is still being verified and there is no information on the validity of the outcome variables. However, reliability on suicide attempt was substantial in that percentage agreement was 88.1% and kappa was 0.70 [19,41]. Nevertheless, in this study, the ratio of completed suicide rate to suicide attempt rate was relatively low compared to previous studies [42], possibly suggesting a lack of validity of outcome variables. Reliability, validity, and correlations with clinical measures of suicide attempt and depressive symptom need to be established in the future. Eighth, as the 12-year study period included only three political regimes, it could be too short to evaluate the impact of political regimes, making it necessary to use longer-term longitudinal data in future research. Ninth, although suicide attempt is the most influential predictor for suicide [5,6,7], those who did not report a suicide attempt history also were associated with increased risk of completed suicide [42], suggesting that, notwithstanding the association between politics and suicide risk in this study, it does not guarantee the association with completed suicide. Finally, this study only assessed associations between politics and adolescent SR, not causality.

## 5. Conclusions

Despite these limitations, this is the first study to assess contextual associations between politics and adolescent SR adjusted for both contextual and individual covariates. Unlike in previous studies [8,14,15], conservative regimes in Korea were associated with lower adolescent SR, which might be attributed to active implementation of suicide prevention policies in response to public health concerns. Contrary to period effects, conservative regimes were related to negative cohort effects on adolescent SR by possibly increasing learning burdens and intensifying competition. These findings imply that, first, policy communities should continue to advocate for suicide prevention policies, because both conservative and liberal regimes can open policy windows to address public health issues [43] and, second, adolescents need to be encouraged to engage in political participation and to choose political forces with policies favorable to their own mental health. 

## Figures and Tables

**Table 1:** Characteristics of adolescents attending middle- and high-school in 2005-2016 Korea Youth Risk Behavior Web-based Survey (KYRBS).

Individual Covariates	Total	Suicide Attempts		Depressive Symptoms	
	*n* = 829,861	*n* = 33,671 (4.1%_wt_)		*n* = 274,431 (33.1%_wt_)	
Age					
12 years old	60,590	2685	(4.6) ^a^	16,423	(27.7) ^a^
13 years old	144,184	6736	(4.7)	42,116	(29.5)
14 years old	145,363	6577	(4.6)	45,257	(31.4)
15 years old	143,456	5924	(4.1)	47,384	(33.0)
16 years old	140,241	5134	(3.6)	48,891	(34.6)
17 years old	135,077	4571	(3.3)	49,894	(36.6)
18 years old	60,950	2044	(3.3)	24,466	(40.1)
Sex					
Male	428,334	12,965	(3.0)	120,382	(28.4)
Female	401,527	20,706	(5.2)	154,049	(38.4)
Academic performance					
High	99,239	3326	(3.4)	27,129	(27.5)
Upper middle	210,729	6453	(3.1)	62,595	(29.7)
Middle	212,743	7016	(3.3)	67,728	(31.9)
Lower middle	211,073	9783	(4.6)	76,416	(36.3)
Low	96,077	7093	(7.3)	40,563	(42.5)
Household economic status					
High	57,559	2760	(4.9)	17,043	(30.1)
Upper middle	211,017	7214	(3.5)	62,654	(29.9)
Middle	369,119	12,228	(3.3)	115,635	(31.5)
Lower middle	150,482	7455	(4.9)	58,673	(38.8)
Low	41,684	4014	(9.7)	20,426	(49.5)
Residential area					
Non-capital non-metropolitan area	311,350	13,069	(4.0)	103,752	(32.7)
Non-capital metropolitan area	221,985	8625	(3.8)	72,985	(32.3)
Capital area	296,526	11,977	(4.2)	97,694	(33.8)
Tobacco use					
Non-user	629,554	18,977	(3.0)	188,009	(30.0)
Past user	111,088	6217	(5.6)	44,185	(39.8)
Current user	47,470	4637	(9.7)	22,109	(46.5)
Daily user	41,749	3840	(9.0)	20,128	(48.0)
Alcohol use					
Non-user	409,592	10,739	(2.6)	107,029	(26.3)
Past user	246,134	10,094	(4.1)	88,599	(36.0)
Current user	171,682	12,262	(7.1)	77,351	(44.9)
Daily user	2453	576	(22.5)	1452	(58.1)
Vigorous physical activity					
None	234,601	842	(4.1)	81,583	(34.7)
1-2 days a week	314,743	12,302	(3.9)	104,274	(33.1)
3-4 days a week	169,398	6848	(4.1)	53,616	(31.9)
5 or more days a week	111,119	4679	(4.2)	34,958	(31.8)
Body mass index					
Normal weight	664,860	26,618	(4.0)	220,147	(33.2)
Underweight	53,645	2307	(4.3)	17,998	(33.7)
Overweight	34,726	1815	(5.3)	12,103	(34.8)
Obesity	76,630	2931	(3.8)	24,183	(31.7)
Healthy diet					
None	3116	300	(9.2)	1166	(37.3)
Less than once a day	757,955	30,462	(4.0)	252,214	(33.3)
Once or more a day	68,790	2909	(4.3)	21,051	(30.9)
Unhealthy diet					
None	109,733	4131	(3.8)	32,420	(29.6)
Less than once a day	713,124	28,710	(4.0)	238,767	(33.6)
Once or more a day	7004	830	(12.4)	3244	(46.7)
Stress level					
Low	481,824	8062	(1.7)	94,769	(19.8)
High	348,037	25,609	(7.3)	179,662	(51.6)
Sleep sufficiency					
Sufficient	223,724	5653	(2.5)	51,418	(23.2)
Insufficient	606,137	28,018	(4.6)	223,013	(36.8)
Depressive symptom					
No	555,430	6042	(1.1)	–	
Yes	274,431	27,629	(10.0)		
Suicidal thought					
No	681,173	2390	(0.3)	–	
Yes	148,688	31,281	(20.8)		

^a^ Unweighted frequency (weighted percentage).

**Table 2:** Associations between political determinants and suicide risk among adolescents attending Korean middle- and high-schools.

Variables	Suicide Attempts OR (95% CI) ^a^		Depressive Symptoms OR (95% CI) ^a^	
Political contextual variables				
Presidency (ref = liberal)				
1st conservative	0.58	(0.47–0.73)	0.71	(0.53–0.93)
2nd conservative	0.76	(0.63–0.91)	0.70	(0.56–0.87)
CEE_NC_ (ref = democratic/liberal)				
Liberal/conservative	1.37	(0.92–2.04)	1.03	(0.96–1.10)
Conservative	2.51	(1.49–4.22)	1.15	(1.04–1.27)
CEE_R_ (ref = liberal)				
1st conservative	1.35	(0.99–1.85)	1.10	(1.04–1.17)
2nd conservative	1.77	(1.03–3.05)	1.15	(1.04–1.27)
Contextual covariates				
Real household final consumption expenditure per capita	0.62	(0.56–0.70)	0.94	(0.86–1.03)
Percentage change of house price index	1.01	(1.00–1.02)	1.02	(1.01–1.04)
College enrolment rate	1.07	(1.03–1.11)	1.14	(1.09–1.20)
Adolescent crime rate	1.10	(1.04–1.17)	1.02	(0.95–1.11)
Labor income share	0.89	(0.86–0.93)	0.95	(0.91–1.00)
Individual covariates				
Age (ref = 18 years old)				
12 years old	1.15	(0.99–1.33)	0.76	(0.72–0.80)
13 years old	1.15	(1.00–1.32)	0.80	(0.77–0.84)
14 years old	1.16	(1.04–1.30)	0.85	(0.82–0.88)
15 years old	1.11	(1.02–1.22)	0.87	(0.84–0.90)
16 years old	1.02	(0.95–1.10)	0.88	(0.85–0.90)
17 years old	1.00	(0.94–1.06)	0.93	(0.91–0.95)
Sex (ref = male)				
Female	1.38	(1.34–1.42)	1.61	(1.59–1.62)
Academic performance (ref = middle)				
High	0.99	(0.95–1.04)	0.85	(0.84–0.87)
Upper middle	0.93	(0.89–0.97)	0.93	(0.92–0.95)
Lower middle	1.08	(1.04–1.11)	1.10	(1.08–1.11)
Low	1.28	(1.23–1.34)	1.19	(1.17–1.22)
Household economic status (ref = middle)				
High	1.51	(1.44–1.59)	1.22	(1.19–1.24)
Upper middle	1.10	(1.06–1.14)	1.10	(1.08–1.11)
Lower middle	1.05	(1.02–1.09)	1.20	(1.18–1.21)
Low	1.41	(1.35–1.47)	1.47	(1.44–1.50)
Residential area (ref = non-capital metropolitan area)				
Non-capital non-metropolitan area	1.06	(1.03–1.09)	0.98	(0.97–1.00)
Capital area	1.04	(1.01–1.07)	1.02	(1.01–1.03)
Tobacco use (ref = non-user)				
Past user	1.31	(1.26–1.35)	1.26	(1.24–1.28)
Current user	1.83	(1.75–1.91)	1.46	(1.43–1.49)
Daily user	1.92	(1.83–2.02)	1.40	(1.36–1.43)
Alcohol use (ref = non-user)				
Past user	1.11	(1.07–1.14)	1.28	(1.27–1.30)
Current user	1.40	(1.35–1.45)	1.60	(1.58–1.63)
Daily user	3.54	(3.11–4.02)	2.56	(2.34–2.79)
Vigorous physical activity (ref = none)				
1–2 days a week	1.04	(1.01–1.07)	1.17	(1.15–1.18)
3–4 days a week	1.10	(1.06–1.14)	1.27	(1.25–1.29)
5 or more days a week	1.16	(1.11–1.21)	1.28	(1.26–1.30)
Body mass index (ref = normal weight)				
Underweight	1.10	(1.05–1.16)	0.99	(0.97–1.01)
Overweight	1.01	(0.95–1.07)	0.98	(0.95–1.00)
Obesity	1.02	(0.98–1.07)	0.95	(0.94–0.97)
Healthy diet (ref = none)				
Less than once a day	0.58	(0.50–0.67)	1.01	(0.94–1.10)
Once or more a day	0.65	(0.56–0.76)	1.05	(0.96–1.13)
Unhealthy diet (ref = none)				
Less than once a day	0.96	(0.92–0.99)	1.13	(1.11–1.15)
Once or more a day	1.68	(1.52–1.85)	1.50	(1.42–1.58)
Stress level (ref = low)				
High	1.16	(1.13–1.20)	3.73	(3.69–3.77)
Sleep sufficiency (ref = sufficient)				
Insufficient	1.04	(1.00–1.07)	1.39	(1.37–1.40)
Depressive symptom (ref = no)				
Yes	1.97	(1.91–2.04)	-	
Suicidal thought (ref = no)				
Yes	44.41	(42.45–46.45)	-	
Intercept	0.001	(0.001–0.002)	0.11	(0.09–0.14)

OR, odds ratio; CI, confidence interval; CEE_NC_, political orientation of presidential regime establishing the National Curriculum reflected in college entrance examination; CEE_R_, political orientation of presidential regime establishing college entrance examination system. ^a^ The odds ratios were produced by cross-classified random intercept logistic regression with students without suicide attempts or depressive symptoms as a reference group for the outcome variables.

## Supplementary Materials

The following are available online at https://www.mdpi.com/1660-4601/16/5/874/s1, Figure S1: Major suicide prevention policies in Korea during 2005–2016. NPSP, national plan for suicide prevention, Table S1: Changes in CEE system by Korean political regimes, Table S2: Description of outcome, political, and covariate variables. Table S3: Characteristics of male adolescents attending middle- and high-school in 2005-2016 KYRBS, Table S4: Characteristics of female adolescents attending middle- and high-school in 2005-2016 KYRBS.
