# Supplementary material for: Contextual Association between Political Regime and Adolescent Suicide Risk in Korea: A 12-year Repeated Cross-Sectional Study from Korea
Source: Int J Environ Res Public Health. 2019 Mar 10;16(5):874. doi: 10.3390/ijerph16050874 (PMC6427480; doi:10.3390/ijerph16050874)
Supplement: Supplementary file 1 [file ijerph-16-00874-s001.zip › supplementary-Table.docx]

Supplementary Table S1 Changes in CEE system by Korean political regimes

| Major changes in CEE system | Description |
| --- | --- |
| Amendment of NC | |
| 7th NC | The 7th NC was founded by the conservative government (President Young-sam Kim, 1993-1997) in 1997, which, however, was the first democratic civilian government after 32 years of military autocracy.  The 7th NC shifted the educational paradigm from the authoritarian tradition that emphasized passive learning of government-standardized content in order to nurture human capital for economic growth, to a learner-centred approach to cultivate democratic citizens with an emphasis on creativity, knowledge acquisition, and individuality [24]. |
| 2007 revision | The liberal government’s 2007 revision of NC also followed the orientation of the former democratic government’s educational reform [24].  The 2007 revision was supposed to be applied to mathematics and English for 7th and 10th grade students in 2009, and to all subjects from 2010-2011. |
| 2009 revision | As Lee’s conservative government took over in 2008, 2007 revision was amended before even being implemented.  The 2009 revision focused on enhancing autonomy of individual schools to alleviate learning burden of students by delegating them authority to develop school-based curriculum [25]. Through reinforcement of autonomy, it sought to foster human resources with global competitiveness by providing room for competency learning [24,25].  During 2011-2013, both the 2007 and 2009 revisions of NC were applied to 10th grade students. |
| CEE policies of each presidential regime | |
| Liberal | The liberal Roh administration diversified the modalities of CEE into high-school records, a college-administered exam, and CSAT starting in the 2008 academic year [18,26,27]. |
| 1st conservative | The first conservative government intensified autonomy of colleges in diversifying CEE and facilitated the admission officer system with the value of “excellence in education,” contrary to the “equality in education” of the previous liberal regime [26,27]. |
| 2nd conservative | The second conservative regime simplified the exam types of CEE, because the former government’s intense diversification of CEE had increased students’ CEE preparation burden, but followed the basic concept of the prior CEE policies that placed weight on diverse academic performance of a candidate rather than on a single CSAT score [27]. |

CEE, college entrance examination; NC, National Curriculum; CSAT, College Scholastic Ability Test.

Supplementary Table S2 Description of outcome, political, and covariate variables

| Variables | Description |
| --- | --- |
| Suicide risk [19] |  |
| Suicide attempt | Attempted suicide in the last 12 months: yes, no |
| Depressive symptom | Feelings of sadness or hopelessness that interfered with one’s everyday life for at least two weeks in the last 12 months: yes, no |
| Political determinants |  |
| Presidency | Presidential term: liberal (2005-2007), 1st conservative (2008-2012), 2nd conservative (2013-2016) |
| CEE_NC_ | NCs applied to CEE: democratic/liberal (grade cohort 1-7), liberal/conservative (8-10), conservative (11-16) |
| CEE_R_ | CEE system by presidential regime: liberal (grade cohort 1-2), 1st conservative (3-8), 2nd conservative (9-16) |
| Contextual covariates [20] |  |
| Economic factors |  |
| Real household final consumption expenditure per capita | Average per capita consumption expenditure of households and private non-profit organizations in constant 2010 prices on final products (gross domestic product) in national accounts |
| Percentage change of house price index | Annual percentage change of house price index assuming that house sales price in June 2015 was 100 |
| Social factors |  |
| College enrolment rate | Percentage of 18 to 21 years old population enrolled in college (bachelor’s or equivalent level) |
| Adolescent crime rate [21] | Number of crimes per 1,000 adolescents under 19 years old |
| Income inequality factor |  |
| Labour income share | Share of wages in national income (sum of wages and operating surplus) |
| Individual covariates [19] |  |
| Sociodemographic factors |  |
| Age | Self-reported age: 12 to 18 years |
| Sex | Self-reported sex: male, female |
| Academic performance | Self-rated school academic achievement in the last 12 months: high, upper middle, middle, lower middle, low |
| Household economic status | Perceived household economic status: high, upper middle, middle, lower middle, low |
| Residential area | Area where one resides: capital area (Seoul, Gyeonggi, Incheon, where about half of the Korean population lives), non-capital metropolitan area (metropolitan cities other than capital area), and non-capital non-metropolitan area (non-metropolitan provinces other than capital area and metropolitan cities) |
| Health behavioural factors |  |
| Tobacco use | Experience of cigarette smoking: non-user (never having smoked), past user (ever smoked, but not in the last 30 days), current user (not smoking every day), daily user (smoking every day) |
| Alcohol use | Experience of alcohol drinking: non-user (never having drunk), past user (ever drank, but not in the last 30 days), current user (not drinking every day), daily user (drinking every day) |
| Vigorous physical activity | Number of days doing vigorous physical activity (e.g., jogging, climbing, heavy lifting) in the last 7 days: 0, 1-2, 3-4, and 5 or more days a week) |
| Body mass index | Self-reported weight (kg) divided by self-reported height (m) squared: normal, underweight, overweight, obesity |
| Healthy diet | Frequency of milk, fruits, and vegetables intake in the last 7 days: none, less than once, once or more a day |
| Unhealthy diet | Frequency of soda and fast food intake in the last 7 days: none, less than once, once or more a day |
| Mental health factors |  |
| Stress level | Level of stress usually felt: high (very much, much), low (somewhat, little, never) |
| Sleep sufficiency | Amount of sleep enough to recover from fatigue in the last 7 days: insufficient (very insufficient, insufficient), sufficient (neither sufficient nor insufficient, sufficient, very sufficient) |
| Suicidal thought | Serious thought of committing suicide in the last 12 months: yes, no   - Suicidal thought and depressive symptom were used as covariates when outcome was suicide attempt. |

CEE, college entrance examination; NC, National Curriculum.

Supplementary Table S3 Characteristics of male adolescents attending middle- and high-school in 2005-2016 KYRBS

| **Individual covariates** | **Total** |  | **Suicide attempt** | |  | **Depressive symptom** | |
| --- | --- | --- | --- | --- | --- | --- | --- |
|  | **n = 428,334** |  | **n = 12,965 (3.0%_wt_)** | |  | **n = 120,382 (28.4%_wt_)** | |
| Age |  |  |  |  |  |  |  |
| 12 years old | 31,328 |  | 903 | (2.9) |  | 7,067 | (23.3) |
| 13 years old | 75,592 |  | 2,303 | (3.1) |  | 18,065 | (24.3) |
| 14 years old | 75,678 |  | 2,394 | (3.2) |  | 19,748 | (26.5) |
| 15 years old | 74,598 |  | 2,352 | (3.1) |  | 21,185 | (28.4) |
| 16 years old | 71,798 |  | 2,045 | (2.8) |  | 21,525 | (30.0) |
| 17 years old | 67,890 |  | 1,968 | (2.8) |  | 21,716 | (31.9) |
| 18 years old | 31,450 |  | 1,000 | (3.2) |  | 11,076 | (35.5) |
| Academic performance |  |  |  |  |  |  |  |
| High | 55,801 |  | 1,729 | (3.2) |  | 13,662 | (24.8) |
| Upper middle | 106,156 |  | 2,439 | (2.3) |  | 27,114 | (25.6) |
| Middle | 107,716 |  | 2,641 | (2.5) |  | 29,055 | (27.2) |
| Lower middle | 106,287 |  | 3,370 | (3.1) |  | 32,077 | (30.7) |
| Low | 52,374 |  | 2,786 | (5.3) |  | 18,474 | (35.8) |
| Household economic status | |  |  |  |  |  |  |
| High | 36,665 |  | 1,644 | (4.5) |  | 9,910 | (27.7) |
| Upper middle | 113,494 |  | 2,888 | (2.6) |  | 28,850 | (25.8) |
| Middle | 181,425 |  | 4,280 | (2.3) |  | 47,713 | (26.7) |
| Lower middle | 74,018 |  | 2,411 | (3.2) |  | 24,231 | (32.7) |
| Low | 22,732 |  | 1,742 | (7.6) |  | 9,678 | (43.4) |
| Residential area |  |  |  |  |  |  |  |
| Non-capital non-metropolitan area | 161,083 |  | 5,157 | (3.1) |  | 45,629 | (28.1) |
| Non-capital metropolitan area | 117,682 |  | 3,485 | (2.9) |  | 33,260 | (27.7) |
| Capital area | 149,569 |  | 4,323 | (3.0) |  | 41,493 | (28.9) |
| Tobacco use |  |  |  |  |  |  |  |
| Non-user | 294,436 |  | 6,426 | (2.2) |  | 70,968 | (24.5) |
| Past user | 69,507 |  | 2,331 | (3.3) |  | 22,786 | (32.9) |
| Current user | 32,252 |  | 2,009 | (6.1) |  | 12,768 | (39.8) |
| Daily user | 32,139 |  | 2,199 | (6.6) |  | 13,860 | (43.3) |
| Alcohol use |  |  |  |  |  |  |  |
| Non-user | 200,393 |  | 4,175 | (2.1) |  | 43,678 | (22.2) |
| Past user | 128,149 |  | 3,531 | (2.7) |  | 37,779 | (29.6) |
| Current user | 97,795 |  | 4,818 | (4.9) |  | 37,800 | (38.9) |
| Daily user | 1,999 |  | 441 | (20.6) |  | 1,125 | (55.3) |
| Vigorous physical activity |  |  |  |  |  |  |  |
| None | 68,819 |  | 2,213 | (3.3) |  | 19,083 | (27.9) |
| 1-2 days a week | 160,670 |  | 4,530 | (2.8) |  | 44,987 | (28.2) |
| 3-4 days a week | 112,116 |  | 3,254 | (2.9) |  | 31,287 | (28.3) |
| 5 or more days a week | 86,729 |  | 2,968 | (3.4) |  | 25,025 | (29.3) |
| Body mass index |  |  |  |  |  |  |  |
| Normal weight | 337,938 |  | 10,034 | (3.0) |  | 95,218 | (28.5) |
| Underweight | 27,561 |  | 1,027 | (3.7) |  | 7,721 | (28.3) |
| Overweight | 8,959 |  | 289 | (3.2) |  | 2,247 | (25.1) |
| Obesity | 53,876 |  | 1,615 | (3.1) |  | 15,196 | (28.6) |
| Healthy diet |  |  |  |  |  |  |  |
| None | 1,773 |  | 152 | (8.3) |  | 553 | (31.0) |
| Less than once a day | 384,433 |  | 11,362 | (2.9) |  | 108,374 | (28.5) |
| Once or more a day | 42,128 |  | 1,451 | (3.5) |  | 11,455 | (27.8) |
| Unhealthy diet |  |  |  |  |  |  |  |
| None | 47,418 |  | 1,296 | (2.8) |  | 11,380 | (24.4) |
| Less than once a day | 376,274 |  | 11,133 | (2.9) |  | 107,001 | (28.7) |
| Once or more a day | 4,642 |  | 536 | (12.3) |  | 2,001 | (44.3) |
| Stress level |  |  |  |  |  |  |  |
| Low | 276,440 |  | 4,188 | (1.5) |  | 48,972 | (18.0) |
| High | 151,894 |  | 8,777 | (5.7) |  | 71,410 | (47.1) |
| Sleep sufficiency |  |  |  |  |  |  |  |
| Sufficient | 294,731 |  | 10,201 | (2.1) |  | 94,197 | (20.0) |
| Insufficient | 133,603 |  | 2,764 | (3.5) |  | 26,185 | (32.1) |
| Depressive symptom |  |  |  |  |  |  |  |
| No | 120,382 |  | 10,370 | (0.8) |  | – |  |
| Yes | 307,952 |  | 2,595 | (8.5) |  |  |  |
| Suicidal thought |  |  |  |  |  |  |  |
| No | 367,318 |  | 1,110 | (0.3) |  | – |  |
| Yes | 61,016 |  | 11,855 | (19.1) |  |  |  |

Supplementary Table S4 Characteristics of female adolescents attending middle- and high-school in 2005-2016 KYRBS

| **Individual covariates** | **Total** |  | **Suicide attempt** | |  | **Depressive symptom** | |  |
| --- | --- | --- | --- | --- | --- | --- | --- | --- |
|  | **n = 401,527** |  | **n = 20,706 (5.2%_wt_)** | |  | **n = 154,049 (38.4%_wt_)** | |  |
| Age |  |  |  |  |  |  |  |  |
| 12 years old | 29,262 |  | 1,782 | (6.4) |  | 9,356 | (32.3) |  |
| 13 years old | 68,592 |  | 4,433 | (6.5) |  | 24,051 | (35.2) |  |
| 14 years old | 69,685 |  | 4,183 | (6.1) |  | 25,509 | (36.8) |  |
| 15 years old | 68,858 |  | 3,572 | (5.2) |  | 26,199 | (38.0) |  |
| 16 years old | 68,443 |  | 3,089 | (4.4) |  | 27,366 | (39.8) |  |
| 17 years old | 67,187 |  | 2,603 | (3.8) |  | 28,178 | (41.8) |  |
| 18 years old | 29,500 |  | 1,044 | (3.5) |  | 13,390 | (45.6) |  |
| Academic performance |  |  |  |  |  |  |  |  |
| High | 43,438 |  | 1,597 | (3.7) |  | 13,467 | (31.1) |  |
| Upper middle | 104,573 |  | 4,014 | (3.9) |  | 35,481 | (33.9) |  |
| Middle | 105,027 |  | 4,375 | (4.2) |  | 38,673 | (37.0) |  |
| Lower middle | 104,786 |  | 6,413 | (6.1) |  | 44,339 | (42.3) |  |
| Low | 43,703 |  | 4,307 | (9.9) |  | 22,089 | (50.8) |  |
| Household economic status | |  |  |  |  |  |  |  |
| High | 20,894 |  | 1,116 | (5.5) |  | 7,133 | (34.3) |  |
| Upper middle | 97,523 |  | 4,326 | (4.5) |  | 33,804 | (34.8) |  |
| Middle | 187,694 |  | 7,948 | (4.3) |  | 67,922 | (36.4) |  |
| Lower middle | 76,464 |  | 5,044 | (6.6) |  | 34,442 | (45.1) |  |
| Low | 18,952 |  | 2,272 | (12.3) |  | 10,748 | (57.3) |  |
| Residential area |  |  |  |  |  |  |  |  |
| Non-capital non-metropolitan area | 150,267 |  | 7,912 | (5.1) |  | 58,123 | (37.9) |  |
| Non-capital metropolitan area | 104,303 |  | 5,140 | (4.8) |  | 39,725 | (37.5) |  |
| Capital area | 146,957 |  | 7,654 | (5.4) |  | 56,201 | (39.1) |  |
| Tobacco use |  |  |  |  |  |  |  |  |
| Non-user | 335,118 |  | 12,551 | (3.8) |  | 117,041 | (35.0) |  |
| Past user | 41,581 |  | 3,886 | (9.4) |  | 21,399 | (51.6) |  |
| Current user | 15,218 |  | 2,628 | (17.4) |  | 9,341 | (61.1) |  |
| Daily user | 9,610 |  | 1,641 | (17.4) |  | 6,268 | (65.0) |  |
| Alcohol use |  |  |  |  |  |  |  |  |
| Non-user | 209,199 |  | 6,564 | (3.2) |  | 63,351 | (30.5) |  |
| Past user | 117,987 |  | 6,563 | (5.6) |  | 50,820 | (43.2) |  |
| Current user | 73,887 |  | 7,444 | (10.3) |  | 39,551 | (53.4) |  |
| Daily user | 454 |  | 135 | (31.9) |  | 327 | (72.0) |  |
| Vigorous physical activity |  |  |  |  |  |  |  |  |
| None | 165,782 |  | 7,629 | (4.5) |  | 62,500 | (37.6) |  |
| 1-2 days a week | 154,073 |  | 7,772 | (5.2) |  | 59,287 | (38.5) |  |
| 3-4 days a week | 57,282 |  | 3,594 | (6.4) |  | 22,329 | (39.2) |  |
| 5 or more days a week | 24,390 |  | 1,711 | (7.3) |  | 9,933 | (41.1) |  |
| Body mass index |  |  |  |  |  |  |  |  |
| Normal weight | 326,922 |  | 16,584 | (5.1) |  | 124,929 | (38.2) |  |
| Underweight | 26,084 |  | 1,280 | (4.9) |  | 10,277 | (39.6) |  |
| Overweight | 25,767 |  | 1,526 | (6.1) |  | 9,856 | (38.3) |  |
| Obesity | 22,754 |  | 1,316 | (5.8) |  | 8,987 | (39.6) |  |
| Healthy diet |  |  |  |  |  |  |  |  |
| None | 1,343 |  | 148 | (10.5) |  | 613 | (46.1) |  |
| Less than once a day | 373,522 |  | 19,100 | (5.1) |  | 143,840 | (38.6) |  |
| Once or more a day | 26,662 |  | 1,458 | (5.7) |  | 9,596 | (36.0) |  |
| Unhealthy diet |  |  |  |  |  |  |  |  |
| None | 62,315 |  | 2,835 | (4.6) |  | 21,040 | (33.8) |  |
| Less than once a day | 336,850 |  | 17,577 | (5.2) |  | 131,766 | (39.2) |  |
| Once or more a day | 2,362 |  | 294 | (12.5) |  | 1,243 | (51.6) |  |
| Stress level |  |  |  |  |  |  |  |  |
| Low | 205,384 |  | 3,874 | (1.9) |  | 45,797 | (22.4) |  |
| High | 196,143 |  | 16,832 | (8.6) |  | 108,252 | (55.2) |  |
| Sleep sufficiency |  |  |  |  |  |  |  |  |
| Sufficient | 311,406 |  | 17,817 | (3.2) |  | 128,816 | (28.0) |  |
| Insufficient | 90,121 |  | 2,889 | (5.8) |  | 25,233 | (41.4) |  |
| Depressive symptom |  |  |  |  |  |  |  |  |
| No | 154,049 |  | 17,259 | (1.4) |  | – |  |  |
| Yes | 247,478 |  | 3,447 | (11.3) |  |  |  |  |
| Suicidal thought |  |  |  |  |  |  |  |  |
| No | 313,855 |  | 1,280 | (0.4) |  | – |  |  |
| Yes | 87,672 |  | 19,426 | (22.1) |  |  |  |  |
