# Supplementary figures and images for: Contextual Association between Political Regime and Adolescent Suicide Risk in Korea: A 12-year Repeated Cross-Sectional Study from Korea
Source: Int J Environ Res Public Health. 2019 Mar 10;16(5):874. doi: 10.3390/ijerph16050874 (PMC6427480; doi:10.3390/ijerph16050874)

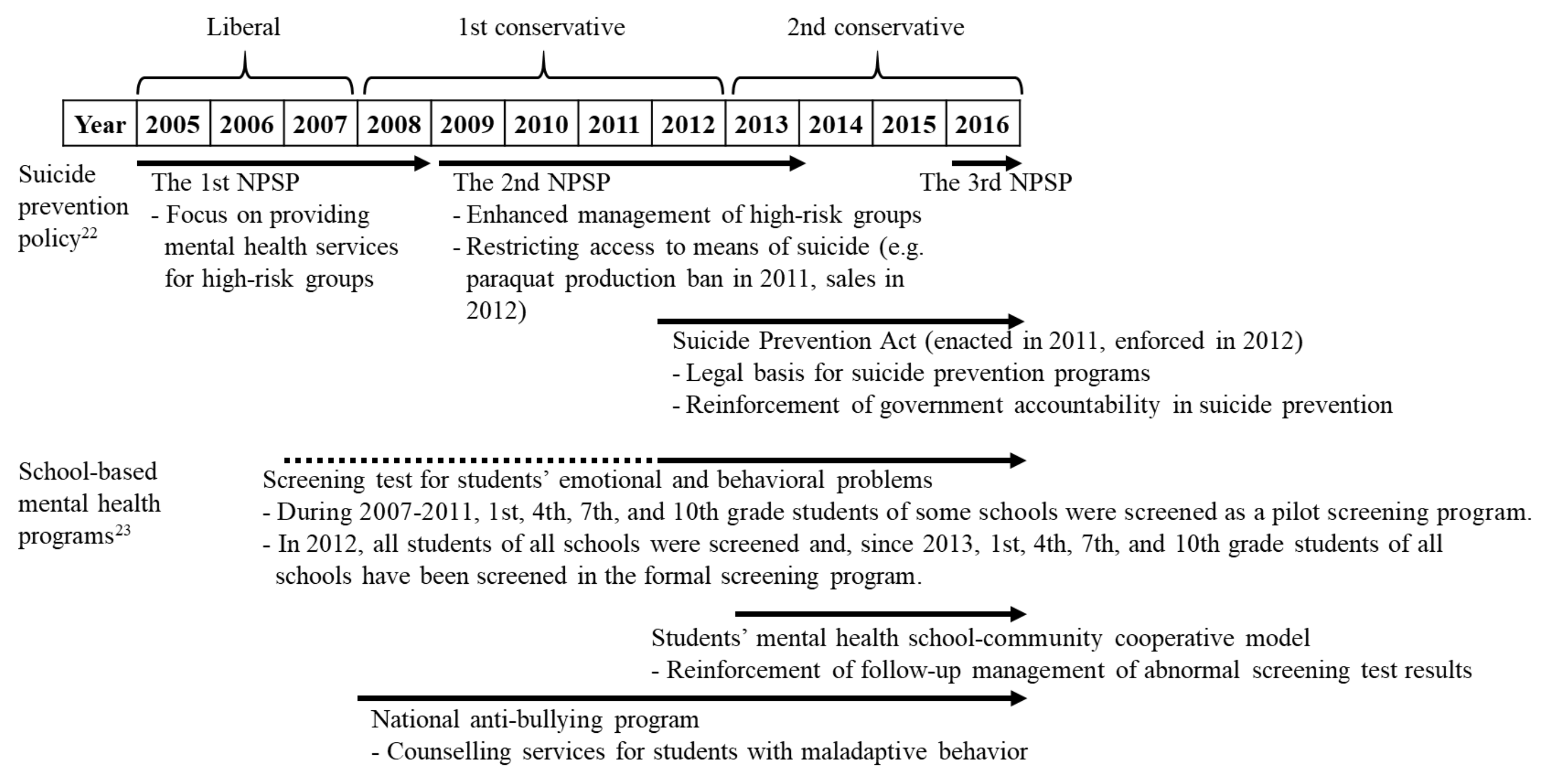

Supplement: Supplementary file 1 [file ijerph-16-00874-s001.zip › supple-Figure.tif]
